# ING5 suppresses breast cancer progression and is regulated by miR-24

**DOI:** 10.1186/s12943-017-0658-z

**Published:** 2017-05-10

**Authors:** Shufang Cui, Xin Liao, Chao Ye, Xin Yin, Minghui Liu, Yeting Hong, Mengchao Yu, Yanqing Liu, Hongwei Liang, Chen-Yu Zhang, Xi Chen

**Affiliations:** 1 State Key Laboratory of Pharmaceutical Biotechnology, Collaborative Innovation Center of Chemistry for Life Sciences, Jiangsu Engineering Research Center for MicroRNA Biology and Biotechnology, NJU Advanced Institute for Life Sciences (NAILS), School of life sciences, Nanjing University, Nanjing, Jiangsu 210046 China; 2 Beihai Marine Station, Evo-devo Institute, School of Life Sciences, Nanjing University, 22 Hankou Road, Nanjing, Jiangsu 210093 China; 3 Department of Exercise and Heath, Nanjing Sport Institute, 8 Linggusi Road, Nanjing, Jiangsu 210014 China

**Keywords:** ING5, microRNA, miR-24, Breast cancer, Proliferation, Invasion, Apoptosis

## Abstract

**Background:**

The inhibitor of growth (ING) gene family of tumor suppressors is involved in multiple cellular functions such as cell cycle regulation, apoptosis, and chromatin remodeling. ING5 is a new member of the ING family whose function and regulation remain largely unknown.

**Methods:**

Quantitative real-time PCR and western blot were used to examine the expression levels of ING5 in breast cancer tissues. The miRNAs that potentially targeted ING5 were determined by bioinformatics analysis and luciferase reporter assay. Cell viability assay, transwell invasion and apoptosis assay were used to characterize the changes induced by overexpressing or knocking down miR-24 or ING5. Hematoxylin and eosin (H&E) staining and immunohistochemical staining for ING5 and Ki-67 were used for xenograft assays in BALB/c nude mice.

**Results:**

We showed that the ING5 protein rather than the mRNA, was significantly downregulated in breast cancer tissues. We also investigated the potential function of ING5 in breast tumorigenesis and found that ING5 suppressed the proliferation and invasion of breast cancer cells and promoted their apoptosis. Furthermore, we explored the molecular mechanisms accounting for the dysregulation of ING5 in breast cancer cells and identified an oncomiR, miR-24, as a direct upstream regulator of ING5. We revealed that miR-24 had the opposite effects to those of ING5 on breast cancer cells and could accelerate xenografted tumor growth in vivo.

**Conclusion:**

Our findings uncover the tumor-suppressive role of ING5 and the regulatory pathway of ING5 in breast cancer and may provide insights into the molecular mechanisms of breast carcinogenesis.

**Electronic supplementary material:**

The online version of this article (doi:10.1186/s12943-017-0658-z) contains supplementary material, which is available to authorized users.

## Background

Cancer is a complicated genetic disease trigged by cells that have accumulated multiple mutations that finally bestow malignant characteristics. Loss or inactivation of tumor suppressor genes, resulting from chromosomal deletion, mutation or hypermethylation, causes immortality of cancer cells [1]. The inhibitor of growth (ING) gene family was identified as an important group of tumor suppressor proteins due to their critical role for the initiation, promotion and development of human cancers [2]. The ING gene family includes five memebers (ING1, ING2, ING3, ING4 and ING5). All ING proteins share a highly conserved carboxy-terminal plant homeodomain (PHD) and regulate several cellular functions relevant to antitumor protection, such as cell cycle restriction, chromatin remodeling, senescence, apoptosis, autophagy and DNA repair.

ING5 is a novel member of the ING family whose fundamental role in tumor suppression has only recently been investigated. ING5 contains a PHD-finger, which is a common motif in proteins involved in chromatin remodeling [3]. ING5 protein can interact with p53 and is involved in the p53-dependent regulatory pathway. Through this pathway and other mechanisms, ING5 induces apoptosis, differentiation and autophagy and decreases proliferation, invasion, metastasis and tumor formation by cancer cells [4–6]. In addition, loss or downregulation of ING5 expression has been frequently observed in different cancer types, including head and neck squamous cell cancer [7], colorectal cancer [6], gastric cancer [8] and oral squamous cell carcinoma [9]. In breast cancer, ING5 has been found to be downregulated and efficiently inhibited the epithelial-mesenchymal transition of cancer cells [10]. However, while the important role of ING5 as a potent tumor suppressor in the progression of human cancers has been frequently documented, the molecular mechanism accounting for the loss expression and dysfunction of ING5 in tumorigenesis is largely unknown and deserves further investigation.

MicroRNAs (miRNAs) are a family of small, non-coding RNAs that play an important role in the regulation of gene expression at the post-transcriptional level. miRNAs bind to complementary sequences in the 3’-untranslated regions (3’-UTRs) of target mRNAs to induce mRNA degradation or translational repression of the target genes. Studies over the past decade have revealed aberrant expression of miRNAs in human cancers as novel biomarkers. In principle, miRNAs can function as oncomiRs or tumor-suppressive miRNAs via targeting tumor suppressor genes or oncogenes, whose dysregulation and dysfunction lead to the misbehavior of cancer cells (e.g., malignant proliferation, invasion and metastasis). miR-24 is one of the most well-known miRNAs correlated with tumorigenesis. Overexpression of miR-24 has been found in a variety of human cancers, including pancreatic cancer [11], non-small cell lung cancer [12], hepatocellular carcinoma [13] and gastric cancer [14]. However, the latent molecular mechanisms through which miR-24 is involved in the development and progression of breast cancer remain to be fully elucidated.

In the present study, we performed a systematic analysis of ING5 expression in breast cancer and identified ING5 as a direct target of miR-24. Furthermore, we showed that miR-24 promoted the proliferation and invasion of, but suppressed the apoptosis of, breast cancer cells in vitro and accelerated xenografted tumor growth in vivo, probably via negatively regulating ING5.

## Methods

### Cell lines and tissues samples

The human breast cancer cell lines (MCF-7 and MDA-MB-231) were purchased from the Shanghai Institute of Cell Biology, Chinese Academy of Sciences (Shanghai, China). The cells were cultured in DMEM medium (Gibco, Carlsbad, CA, USA) supplemented with 10% fetal bovine serum (FBS, Gibco) in a humidified incubator at 37 °C with 5% CO_2_. Paired breast cancer and adjacent noncancerous tissue samples were obtained from patients who were undergoing surgical procedures at the Affiliated Drum Tower Hospital of Nanjing University Medical School (Nanjing, China). Written consent was obtained from all of the patients and all aspects of this project were approved by the Clinical Research Ethics Committee from the Affiliated Drum Tower Hospital of Nanjing University Medical School. Tissue specimens were immediately frozen in liquid nitrogen after resection and stored at −80 °C. For clinical features of the patients, see Table 1.

**Table 1:** Clinical features of breast cancer patients

Case no.	Clinical history	Gender	Age (years)	TNM stage
BC #1	IDC	Female	57	II
BC #2	IDC	Female	56	II
BC #3	IDC	Female	61	II-III
BC #4	IDC	Female	55	III
BC #5	IDC	Female	56	I-II
BC #6	IDC	Female	48	II
BC #7	IDC	Female	55	II-III
BC #8	IDC	Female	50	II-III

### RNA isolation and quantitative RT-PCR

RNA isolation and hydrolysis probe-based quantitative RT-PCR (qRT-PCR) were performed as previously described [15]. For the quantification of miR-24 and U6 snRNA, 0.2 μg of total RNA obtained from cultured cells or tissues was reverse-transcribed to cDNA using AMV reverse transcriptase (TaKaRa, Dalian, China). The reaction mixture was incubated at 16 °C for 30 min, 42 °C for 30 min, and 85 °C for 5 min.qRT-PCR was carried out with a TaqMan PCR kit on an Applied Biosystems 7300 Sequence Detection System (Applied Biosystems). The real-time PCR cycles consisted of a pre-denaturation step at 95 °C for 5 min, followed by 40 cycles of 95 °C for 15 s and 60 °C for 1 min. After the reactions, the cycle threshold (C_T_) data were determined using default threshold settings, and the average C_T_ was calculated from triplicate PCRs. C_T_ values of miR-24 were normalized to U6 snRNA, and the relative levels of miR-24 were determined using the formula 2^-ΔΔCT^, in which ΔΔC_T_ = (C_T miR-24_ – C_T U6_) _target_ - (C_T miR-24_ – C_T U6_) _control_.

For the quantification of ING5 and GAPDH mRNA, 1 μg of total RNA was reverse-transcribed to cDNA using Oligo d(T) primer (TaKaRa, Dalian, China). qRT-PCR was then performed using SYBR Green dye (Invitrogen) and specific primers for ING5 and GAPDH. The C_T_ values were determined by setting a fixed threshold. The relative levels of ING5 mRNA were normalized to GAPDH using the 2^-ΔΔCT^ method as described above.

The sequences of the primers used for amplification were as follows:

- ING5 (sense): 5′-AAACGAACCCACGTACTGC-3′;
- ING5 (antisense): 5′-TTGCGACACGAATGAAGG-3′;
- GAPDH (sense): 5′-CGAGCCACATCGCTCAGACA-3′;
- GAPDH (antisense): 5′-GTGGTGAAGACGCCAGTGGA-3′.

### miRNA target prediction

TargetScan algorithm (http://genes.mit.edu/targetscan/) was used for searching potential miRNAs that could target ING5.

### miRNA overexpression or knockdown

Lipofectamine 2000 (Invitrogen) was used for transfection in accordance with procedures suggested by the manufacturer. The overexpression of miR-24 was achieved by transfecting cells with a miR-24 mimic (pre-miR-24, a synthetic double-stranded RNA oligonucleotide mimicking the precursor of miR-24), whereas the knockdown of miR-24 was achieved by transfecting cells with a miR-24 inhibitor (anti-miR-24, a chemically modified antisense oligonucleotide designed to target mature miR-24). Scrambled RNAs were used as the negative controls (pre-miR-control and anti-miR-control). Equal amounts of RNAs were transfected when the cells were approximately 70% confluent. The cells were harvested 24 h after the transfection for the isolation of RNA or protein. The MCF-7 cells were also treated with actinomycin D (2 μg/mL, Sigma) and incubated for 8 h to inhibit transcription after 12 h of transfection.

### Protein isolation and western blotting

Tissue samples were processed in a Tissuelyser machine (Jingxin, Shanghai, China). The cells or processed tissue products were lysed in RIPA lysis buffer and the protein supernatants were collected after centrifuging. A BCA protein assay kit (Thermo Scientific, Rockford, IL, USA) was used to calculate the protein concentration. For western blotting, equal amounts of protein samples were separated on SDS-PAGE gels and transferred to a PVDF membrane (Bio-Rad, California, USA). After blocking in 5% skim milk, the membranes were incubated with primary antibodies (ING5, 1:800, Proteintech Group, Inc, IL, USA; GAPDH, 1:2000, Santa Cruz Biotechnology) () at 4 °C overnight. After incubation with goat anti-rabbit secondary antibody (1:2000, Santa Cruz Biotechnology) for 1 h at room temperature, the bands were detected with the SuperSignal West Pico chemiluminescence substrate (Pierce, Thermo Scientific).

### Plasmid construction and siRNA interference assay

A mammalian expression plasmid designed to specifically express the open reading frame (ORF) of human ING5 without the miR-24-targeted 3’-UTR was purchased from Invitrogen. An ING5-HA fusion plasmid which inserted a hemagglutinin (HA) fragment (TACCCTTATGATGTGCCAGATTATGCC) at the C-terminal of the ING5 expression plasmid was purchased from Realgene. The siRNA -targeting human ING5 was designed and synthesized by Invitrogen (sense) 5′-GCGCUUUGAAGCAGAUCUGTT-3′; (antisense) 5′-CAGAUCUGCUUCAAAGCGCTT-3′. The empty plasmid backbone and a scrambled siRNA served as the negative control. For MCF-7 cells seeded in 6-well plates, equal amount of plasmid (0.02 μg) or siRNA (200 pmol) were transfected.

### Luciferase reporter assay

The human ING5 3’-UTR with a wild-type or a mutant miR-24 target sequence was inserted into the p-MIR-reporter plasmid (Ambion) to create p-MIR-luc-ING5 WT or p-MIR-luc-ING5 Mut, respectively. The desired constructs were confirmed by sequencing. The sequence that interacts with the seed sequence of miR-24 was mutated (from CTGAGCC to GACTCGG). For the luciferase reporter assay, MCF-7 cells were seeded into 24-well plates and transfected with a mixture of 0.2 μg p-MIR-luc-ING5 WT or Mut, 0.1 μg β-galactosidase (β-gal) expression plasmid (Ambion) and equal amounts (20 pmol) of RNAs. The β-gal plasmid was used as a transfection control. The luciferase activity was analyzed 24 h after transfection using luciferase assay kits (Promega, Madison, WI, USA).

### Cell viability assay

MCF-7 cells were plated at 1 × 10^3^ cells per well in 96-well plates in 100 μL DMEM medium supplemented with 1% FBS. Cells were then collected at 12, 24, 36, 48, 60, 72, 96 and 120 h after transfection. The Cell Counting Kit-8 (CK04-500, Dojindo) was used to determine the viability of the MCF-7 cells based on the procedures suggested by the manufacturer. Briefly, 10 μL of the provided CCK-8 liquid was added to the test wells and the plates were incubated for 2 h. The absorbance was then measured at a wavelength of 450 nm.

### Transwell invasion assay

For the invasion assay, 2 × 10^4^ transfected MCF-7 cells were suspended in serum-free DMEM and seeded into the upper invasion chamber (BD Biosciences, Bedford, MA) that was pre-coated with a thin layer of Matrigel. DMEM containing 10% FBS was placed into the lower compartment as a chemo-attractant. The cells were allowed to invade for 10 h, and then the cells adhering to the lower Matrigel membrane were fixed 4% paraformaldehyde and stained with 0.1% crystal violet solution at room temperature. The noninvasive cells in the upper chamber were gently scraped out with cotton swabs. The invaded cells were counted in 3 different fields per well under a microscope at 100 × magnification.

### Cell apoptosis assay

The apoptosis of MCF-7 cells was determined using the FITC-Annexin V Apoptosis Detection Kit I (BD Biosciences) based on the procedures provided by the manufacturer. The transfected MCF-7 cells were cultured in serum-free DMEM for 24 h. The collected cells were washed with cold PBS and resuspended in 1 × binding buffer, followed by staining with FITC-Annexin V and propidium iodide (PI) in the dark for 15 min. The apoptotic cells were calculated using a fluorescence-activated cell-sorting (FACS) flow cytometer (BD Biosciences, San Jose, CA, USA).

### Xenograft assays in nude mice

Athymic BALB/c female nude mice (4 weeks old) were purchased from the Model Animal Research Center of Nanjing University (Nanjing, China) and were randomly divided into 3 groups (3 mice per group). MCF-7 cells were infected with a control lentivirus or a lentivirus to overexpress miR-24, or transfected with an ING5 overexpressing plasmid. After treatment, the MCF-7 cells were uniformly suspended in PBS at a final concentration of 1× 10^7^ per mL. For each mouse, 0.1 mL cell suspension solution (1× 10^6^ cells) was subcutaneously injected and all the injection performance was carried out according to the standard protocol. The needle was inserted into the armpit of the right hind leg at a 45 degree angle and a 5 mm depth, midway down. The mice were sacrificed and photographed 28 days post-injection. The xenograft tumors were removed and weighed. Part of tissue was used for extracting total RNA and protein, and the remainder tissue was made into tumor section slides for hematoxylin and eosin (H&E) staining and immunohistochemical staining for ING5 and Ki-67. All animal care and handling procedures were performed in accordance with the National Institutes of Health’s Guide for the Care and Use of Laboratory Animals.

### Statistical analysis

The results are presented as the means ± SE. All of the images shown are representative of at least three independent experiments. qRT-PCR, luciferase reporter assay, cell viability assay, transwell invasion assay and cell apoptosis assay were all performed in triplicate, and each experiment was repeated several times. The differences were considered to be significant at *p* < 0.05 based on Student’s *t*-test.

## Results

### ING5 is a negative regulator of tumorigenesis in breast cancer

**Fig. 1:**
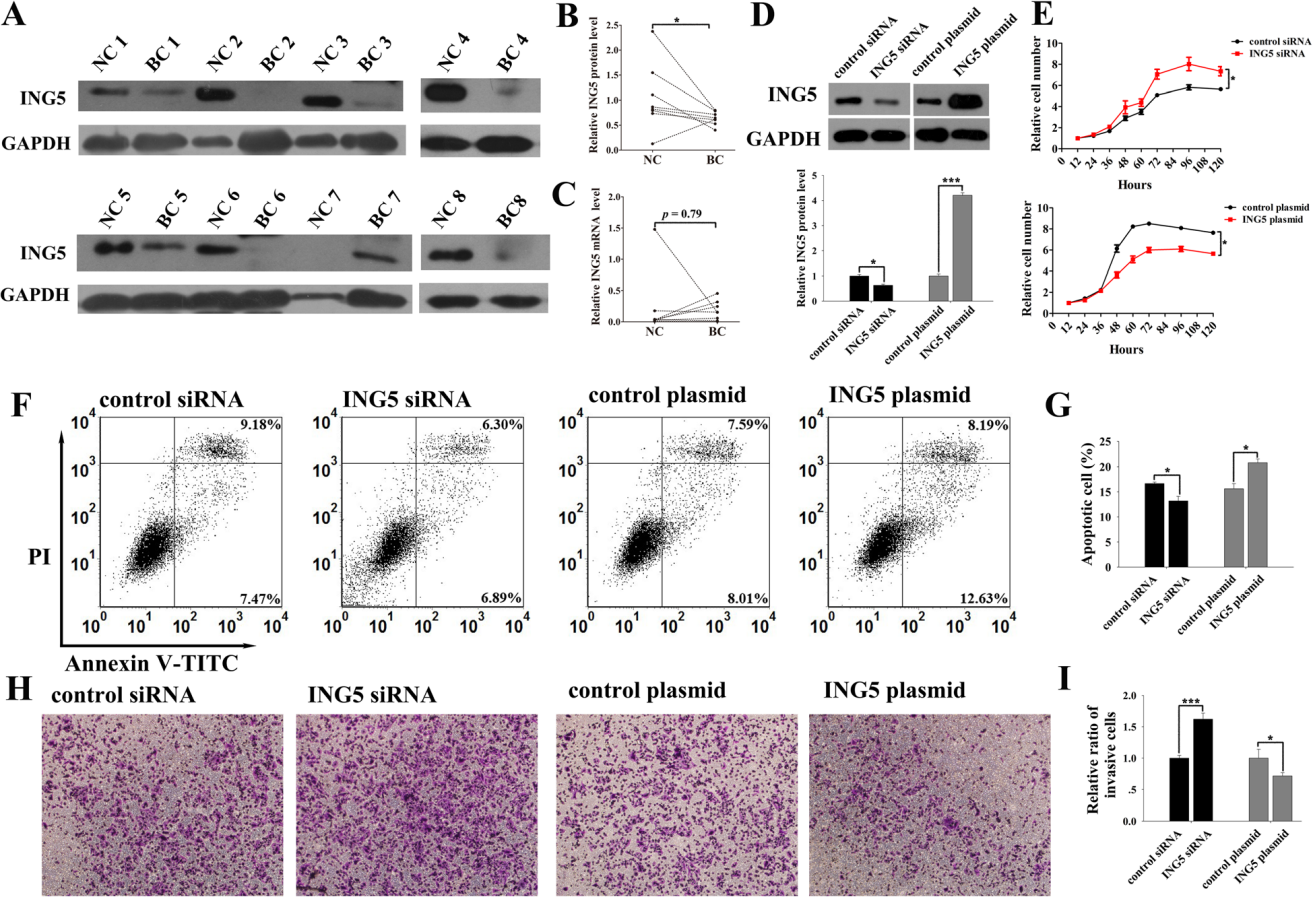
Expression levels of ING5 in breast cancer tissues and its role in breast cancer cells. **a**-**b** Western blot analysis of the expression levels of ING5 protein in 8 pairs of breast cancer (BC) and noncancerous (NC) tissue samples. **a**: representative image of the western blot assay; **b**: quantitative analysis of the western blot assay. **c** qRT-PCR analysis of the expression levels of ING5 mRNA in 8 pairs BC and NC tissue samples. **d** Western blot analysis of the expression levels of ING5 protein in MCF-7 cells treated with control siRNA, ING5 siRNA, control plasmid or an ING5 overexpressing plasmid. *Upper panel*: representative image; *lower panel*: quantitative analysis. **e**-**i** The effects of ING5 on the proliferation, apoptosis and invasion of MCF-7 cells transfected with control siRNA, ING5 siRNA, control plasmid or an ING5 overexpressing plasmid. **e**: the proliferation curves; **f**: representative image of apoptosis assays; **g**: quantitative analysis of apoptosis assays; **h**: representative image of invasion assays; **i**: quantitative analysis of invasion assays. *p < 0.05; ***p < 0.001

First, we measured the expression levels of ING5 protein and mRNA in 8 paired human breast cancer and noncancerous tissues. The ING5 protein levels were significantly lower in breast cancer tissues than in the paired normal tissues (Fig. 1a-b). However, inconsistent alterations of the ING5 mRNA level were observed in breast cancer tissues when compared with the matched noncancerous tissues (Fig. 1c). These results suggest that the ING5 protein rather than the mRNA is downregulated in breast cancer.

To investigate the potential function of ING5 in breast tumorigenesis, we silenced ING5 expression in the MCF-7 breast cancer cell line by transfecting the cells with siRNA directed against ING5. The efficient knockdown of ING5 in the MCF-7 cells is observed (Fig. 1d). Knockdown of ING5 significantly increased the proliferation of the MCF-7 cells (Fig. 1e), whereas the percentage of apoptotic cells was significantly decreased in cells treated with the ING5 siRNA (Fig. 1f-g). Moreover, knockdown of ING5 significantly increased the number of MCF-7 cells that invaded through the Matrigel-coated filter in the transwell chamber (Fig. 1h-i). On the other hand, we enhanced ING5 expression in the MCF-7 cells by transfecting the cells with a human ING5 overexpression plasmid. The efficient overexpression of ING5 in the MCF-7 cells is observed (Fig. 1d). ING5 overexpression stimulated cell apoptosis and inhibited cell proliferation and invasion of the MCF-7 cells (Fig. 1e-i). Taken together, these results suggest that ING5 plays an anti-tumor role in breast cancer cells.

### ING5 is a direct target of miR-24

**Fig. 2:**
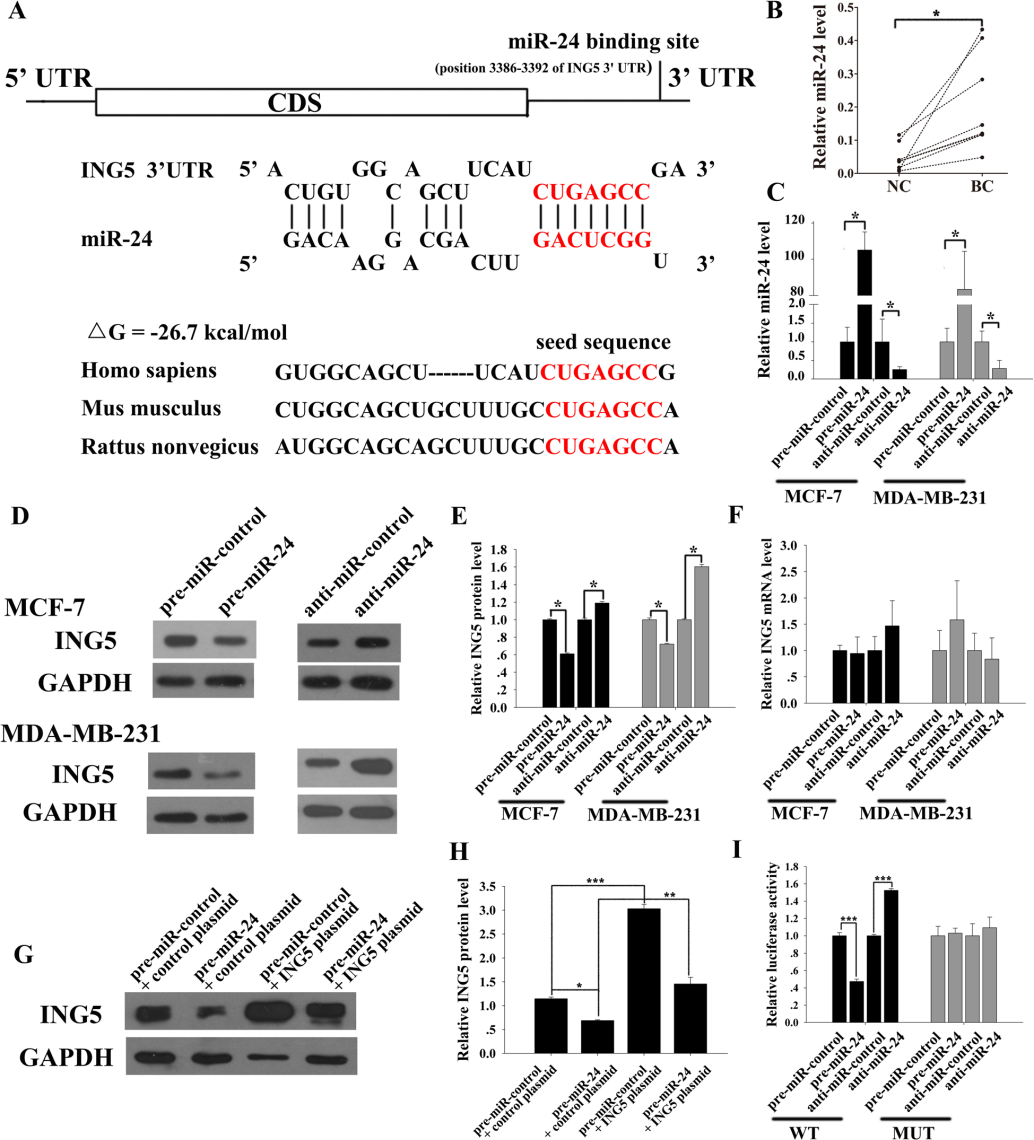
Prediction and validation of ING5 as a direct target of miR-24. **a** Schematic description of the hypothetical duplex formed by the interactions between the binding site in the ING5 3’-UTR (top) and miR-24 (bottom). The seed region of miR-24 and the seed-recognition site in the ING5 3’-UTR are indicated in red. All nucleotides in the seed-recognition site are completely conserved in several species. The predicted free energy value of the hybrid is calculated. **b** qRT-PCR analysis of the expression levels of miR-24 in the same 8 pairs of BC and NC tissue samples. **c**-**f** qRT-PCR and western blot analysis in MCF-7 and MDA-MB-231 cells transfected with equal doses of scrambled negative control mimic (pre-miR-control), miR-24 mimic (pre-miR-24), scrambled negative control inhibitor (anti-miR-control) or miR-24 inhibitor (anti-miR-24). **c**: qRT-PCR analysis of miR-24; **d**: representative image of western blot analysis of ING5 protein; **e**: quantitative analysis of western blot; **f**: qRT-PCR analysis of ING5 mRNA. **g**-**h** Western blot analysis of the expression levels of ING5 protein in MCF-7 cells co-transfected with pre-miR-control plus control plasmid, pre-miR-24 plus control plasmid, pre-miR-control plus an ING5 overexpressing plasmid, or pre-miR-24 plus an ING5 overexpressing plasmid. **g**: representative image; **h**: quantitative analysis. **i** The relative luciferase activities in MCF-7 cells transfected with wild-type or mutant ING5 luciferase plasmid and with equal doses of pre-miR-control, pre-miR-24, anti-miR-control or anti-miR-24. *p < 0.05; **p < 0.01; ***p < 0.001

The disparity between the levels of ING5 protein and ING5 mRNA expression in breast cancer tissues strongly implies that a post-transcriptional mechanism is involved in ING5 regulation in breast cancer. Because miRNAs are well established as potent post-transcriptional regulators of gene expression, the TargetScan computational algorithm was used to identify potential miRNAs that can target ING5. Among the candidate miRNAs, miR-24 was selected for further experimental verification because miR-24 is a well-known oncomiR that has been frequently associated with malignancies [16, 17]. The predicted interaction between miR-24 and the target sequence within the ING5 3’-UTR is illustrated in Fig. 2a. As shown in this figure, there is one conserved miR-24 binding site in the 3’-UTR of the ING5 mRNA sequence. The minimum free energy value of the hybridization is −26.7 kcal/mol, which is well within the range of genuine miRNA-target pairs.

Subsequently, we measured the expression levels of miR-24 in the same 8 pairs of breast cancer tissues and noncancerous tissues. qRT-PCR showed that the miR-24 levels were consistently higher in the cancer tissues (Fig. 2b). Spearman correlation analysis showed that there was a trend of inverse correlation between the miR-24 levels and ING5 protein levels in paired breast cancer samples (Additional file 1: Figure S1).

To elucidate whether miR-24 could be a regulator of ING5 protein expression in breast cancer, we determined ING5 expression levels after the overexpression or the knockdown of miR-24 in human breast cancer MCF-7 and MDA-MB-231 cells. The cellular miR-24 levels increased by approximately 100-fold when the MCF-7 and the MDA-MB-231 cells were transfected with a miR-24 mimic, and these levels dropped to approximately 20% of the normal levels when the cells were treated with a miR-24 inhibitor (Fig. 2c). In parallel with the changes in the miR-24 levels, the expression of the ING5 protein was significantly reduced by the introduction of miR-24, whereas transfection with a miR-24 inhibitor significantly increased the ING5 protein levels in the MCF-7 and the MDA-MB-231 cells (Fig. 2d-e). In contrast, overexpression or knockdown of miR-24 did not affect ING5 mRNA levels (Fig. 2f), implying that miR-24 controlled the expression of ING5 protein at the translational level. To test this hypothesis, MCF-7 cells transfected with miR-24 mimic or inhibitor were treated with actinomycin D to inhibit transcription [18]. The unstable c-myc proto-oncogene was used as a positive control to confirm the efficiency of transcription inhibition (Additional file 2: Figure S2A). Overexpressing or knocking down miR-24 had no effect on the levels of already existed ING5 mRNAs after blocking the production of nascent mRNAs (Additional file 2: Figure S2B), but still significantly decreased the levels of ING5 protein (Additional file 2: Figure S2C-D). These results revealed that miR-24 regulated the expression of ING5 protein at the translational level.

More importantly, co-transfecting the MCF-7 cells with an ING5 overexpression plasmid and a miR-24 mimic significantly increased ING5 expression compared with cells transfected with the miR-24 mimic alone (Fig. 2g-h), suggesting that the expression of miR-24-resistant ING5 plasmid (ING5 ORF without the miR-24-targeted 3’-UTR) was sufficient to rescue the suppression of ING5 by miR-24. To further confirm that induction of exogeneous ING5 could rescue the suppression of ING5 by miR-24, we designed a HA-tagged ING5 plasmid and repeated the above co-transfection experiments. As expected, overexpression of miR-24 did not change the levels of HA polypeptide but significantly decreased the levels of total ING5 protein when co-transfecting the MCF-7 cells with an ING5-HA plasmid and a miR-24 mimic (Additional file 3: Figure S3A-B). These results revealed that miR-24-resistant ING5 plasmid can rescue the repression of endogenous ING5 by miR-24.

Next, we performed a luciferase reporter assay to determine whether the negative regulatory effect that miR-24 exerted on ING5 expression was mediated specifically through the binding of miR-24 to the presumed site in the ING5 3’-UTR. The ING5 3’-UTR harboring the wild type or mutant miR-24 target sequence was inserted downstream of the firefly luciferase gene in a reporter plasmid. The recombinant plasmids were transfected into MCF-7 cells along with a miR-24 mimic, a miR-24 inhibitor or scrambled negative control RNAs. Overexpression of miR-24 significantly suppressed the luciferase activity in MCF-7 cells, whereas inhibition of miR-24 had an opposite effect (Fig. 2i). In contrast, neither the overexpression nor the knockdown of miR-24 changed the luciferase activity of the mutant luciferase reporter (Fig. 2i). In summary, these results suggest that miR-24 directly binds to the 3’-UTR of the ING5 transcript and mediates the post-transcriptional repression of ING5 expression.

### miR-24 and ING5 have opposite effects on breast cancer cell proliferation, invasion and apoptosis

**Fig. 3:**
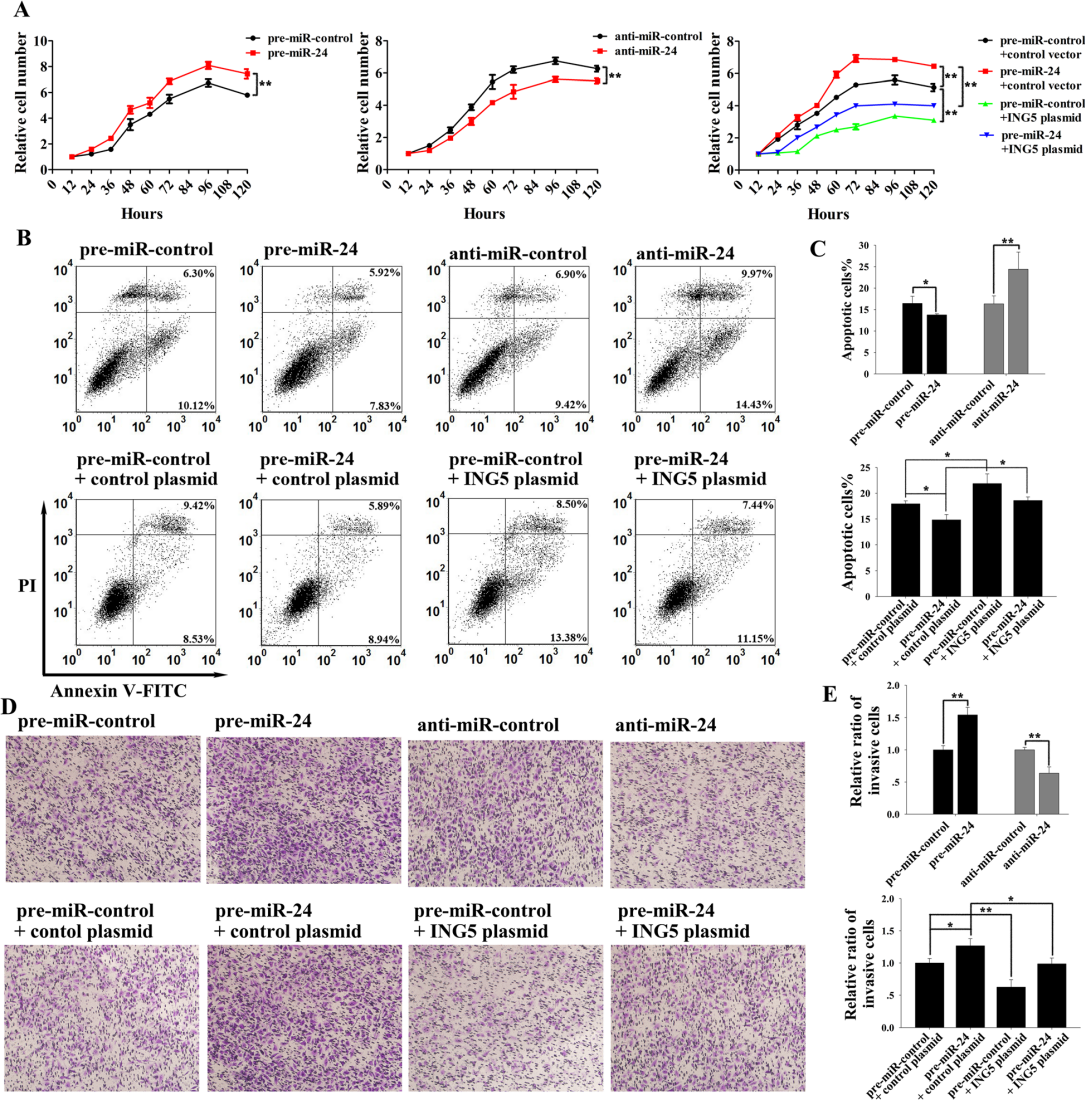
Effects of miR-24 on the proliferation, apoptosis and invasion of breast cancer cells. MCF-7 cells were transfected with pre-miR-control or pre-miR-24, with anti-miR-control or anti-miR-24, or with pre-miR-control plus control plasmid, pre-miR-24 plus control plasmid, pre-miR-control plus an ING5 overexpressing plasmid, or pre-miR-24 plus an ING5 overexpressing plasmid. **a** The proliferation curves of MCF-7 cells. **b** The representative image of apoptosis assays. **c** The quantitative analysis of apoptosis assays. **d** The representative image of transwell invasion assays. **e** The quantitative analysis of transwell invasion assays. *p < 0.05; **p < 0.01

To investigate the biological consequences of the downregulation of ING5 expression caused by miR-24 in breast cancer, we analyzed the effects of miR-24 on cell proliferation, invasion and apoptosis after the overexpression or the knockdown of miR-24 in MCF-7 cells. The MCF-7 cells transfected with the miR-24 mimic showed an increased proliferation rate, whereas the knockdown of miR-24 had the opposite effect on cell proliferation (Fig. 3a). Likewise, the percentage of apoptotic cells was significantly lower in the cells transfected with a miR-24 mimic, whereas the knockdown of miR-24 increased the percentage of apoptotic cells (Fig. 3b-c). Transfecting MCF-7 cells with a miR-24 mimic significantly increased the number of cells invading through a Matrigel-coated filter, whereas the knockdown of miR-24 reduced cell invasion (Fig. 3d-e). These results suggest that miR-24 and ING5 have opposite effects on breast cancer cell proliferation, invasion and apoptosis. Moreover, when compared to cells transfected with the miR-24 mimic, the cells transfected with the miR-24 mimic and the ING5 overexpression plasmid exhibited significantly lower proliferation and invasion rates and higher apoptotic percentage (Fig. 3a-e), suggesting that miR-24-resistant ING5 can attenuate the oncogenic effect of miR-24. Thus, the promotion of cell proliferation and invasion, and the inhibition of cell apoptosis, by knockdown of ING5 were similar to the effects elicited by miR-24 overexpression, further indicating that miR-24 may stimulate cell proliferation and invasion and suppress cell apoptosis by silencing ING5.

### miR-24 decreases ING5 expression and promotes xenografted tumor growth in vivo

**Fig. 4:**
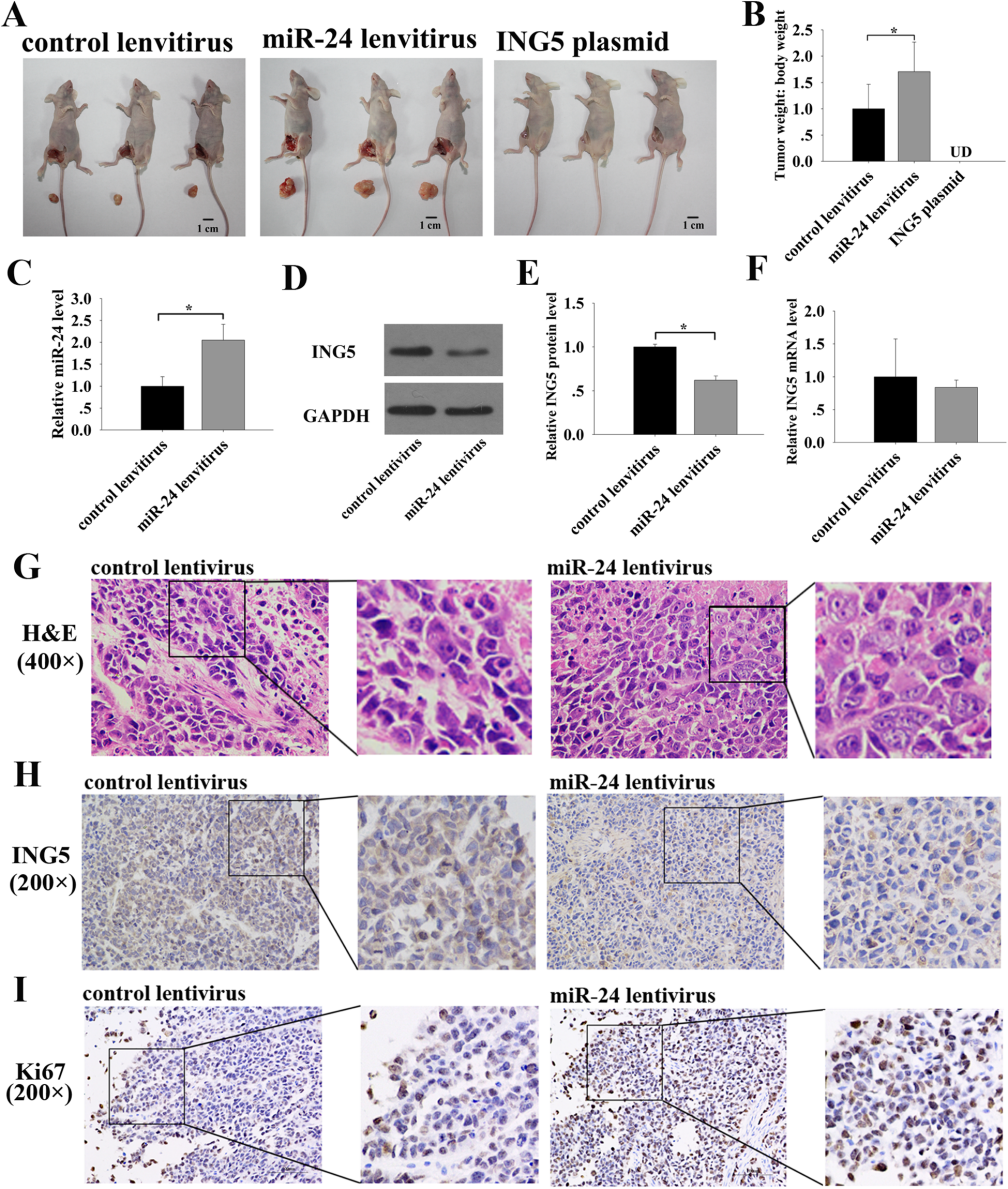
Effects of miR-24 and ING5 on the growth of breast cancer cell xenografts in mice. **a** Representative images of the injected mice and the tumors from the injected mice. **b** Quantitative analysis of the tumor weights. **c** qRT-PCR analysis of the expression levels of miR-24 in the tumors from the injected mice. **d**-**e** Western blot analysis of the expression levels of ING5 protein in the tumors from the injected mice. **d**: representative image; **e**: quantitative analysis. **f** qRT-PCR analysis of the expression levels of ING5 mRNA in the tumors from the injected mice. **g** Representative H&E-stained sections in the tumors from the injected mice. **h** Representative immunohistochemical staining for ING5 in the tumors from the injected mice. **i** Representative immunohistochemical staining for Ki-67 in the tumors from the injected mice. *p < 0.05

Next, we evaluated the effect of miR-24 and ING5 on the growth of breast tumors in a xenograft mouse model. A significant increase in the size and weight of the tumors was observed in the miR-24-overexpressing group compared to the control group (Fig. 4a-b). Strikingly, no tumors were found in the ING5-overexpressing group (Fig. 4a-b). Subsequently, total RNA and protein were extracted from each xenograft and used to evaluate the expression levels of miR-24 and ING5. Tumors from the miR-24-overexpressing group showed a significant increase in the expression of miR-24 compared to tumors from the control group (Fig. 4c). Consequently, reduced ING5 protein levels were observed in the miR-24-overexpressing group compared to the control group (Fig. 4d-e), whereas levels of ING5 mRNA remained unchanged (Fig. 4f). In addition, H&E staining of the xenograft tissues showed more cell mitosis in tumors from the miR-24-overexpressing group than in tumors from the control group (Fig. 4g). Accordingly, immunohistochemical staining for ING5 revealed the presence of lower levels of ING5 in the group injected with miR-24-overexpressing cells (Fig. 4h). Finally, the proliferative activity of the tumor cells was assessed via immunohistochemistry using a mouse monoclonal antibody, Ki-67. The tumor cell proliferation rate, as measured by the percentage of Ki-67-positive tumor cells, was increased in tumors from the miR-24-overexpressing group (Fig. 4i). These results are consistent with the findings of the in vitro assays and firmly validate the oncomiR role of miR-24 in breast tumorigenesis, probably via negatively regulating ING5.

## Discussion

Breast cancer continues to be the leading cause of cancer-related mortality in women worldwide. Despite advances in diagnosis and access to different types of treatment such as surgery, radiotherapy, chemotherapy and hormone therapy, numerous women with breast cancer continue to succumb to this malignancy. Some treatments for breast cancer may cause side effects that persist or appear months or years after treatment. Understanding the underlying mechanisms that regulate the progression of breast cancer is crucial for the development of novel therapeutics. Considerable research efforts should also be performed to develop novel therapeutic strategies to combat breast carcinogenesis.

ING proteins have recently received more attention due to their involvement in the development of multiple tumors [19, 20]. ING5 belongs to the ING tumor suppressor family and aberrant expression of ING5 has been found in several tumors [6–9]. Consistent with these reports, here in we found that the ING5 protein was downregulated in breast cancer tissues compared to normal adjacent tissues. Indeed, downregulation of ING5 is involved in the pathogenesis of breast cancer and some other types of cancer. For example, a genome-wide functional screen by Mendes-Pereira identified ING5 as one of the set of genes whose silencing caused sensitivity to tamoxifen in breast cancer therapy regimens [21]. Furthermore, it has been reported that ING5 may inhibit cancer aggressiveness via prevention of the epithelial-mesenchymal transition in breast cancer and lung cancer [10, 22]. Additionally, tumor-specific mutation and downregulation of ING5 mRNA were found in oral squamous cell carcinoma [9]. These findings validate that ING5 is involved in cancer pathogenesis as a tumor suppressor gene. Likewise, our functional study also supports the anti-tumor role of ING5 in breast cancer cells. Overexpression of ING5 significantly inhibited the proliferation and invasion of breast cancer cells, but stimulated apoptosis, while knockdown of ING5 had the opposite effects on these cellular functions. Epigenetic modification of cancer cells by either silencing oncogenes or reactivating silenced tumor suppressors may sensitize cancer cells to lower doses of conventional cytotoxic agents. For example, the combination of ING1 with a chemical agent can act synergistically to block breast cancer cell growth [23]. Following this theory, it is quite possible that ING5 may also affect breast cancer cell response to chemotherapeutic drugs. Thus, induction of ING5 expression may become an attractive therapeutic strategy for breast cancer.

It is well known that miRNAs interact with the 3’-UTRs of their target mRNAs through imperfect complementary binding and that the targeted transcripts subsequently undergo accelerated turnover and translational repression [24]. As potent post-transcriptional regulators of gene expression, miRNAs may be therefore involved in ING5 regulation and may well explain the disparity between ING5 protein and ING5 mRNA expression in breast cancer tissues. To determine the molecular mechanism underlying ING5 downregulation in breast cancer, we used computational bioinformatics to predict those miRNAs that could target ING5 and identified miR-24. Aberrant expression of miR-24 has been found in multifarious cancer types and miR-24 has both tumor-suppressive and oncogenic properties depending on the cellular context. In breast cancer, it has been shown that miR-24 exhibited great capability for discriminating and monitoring between cancer patients and controls [25, 26]. Moreover, miR-24 played a key role in breast cancer invasion and metastasis [17], suggesting the oncogenic role of miR-24 in breast cancer. In the present study, we found that miR-24 levels were consistently higher in breast cancer tissues. We then experimentally validated ING5 as a novel target of miR-24 by cell transfection and luciferase reporter assays. Furthermore, we provided evidence that induction of miR-24 expression could mimic ING5 suppression, stimulating the proliferation and invasion of breast cancer cells, and suppressing apoptosis. In addition, restoration of ING5 expression could reverse the miR-24-induced cellular phenotypes, suggesting that targeting ING5 is a major mechanism by which miR-24 exerts its oncogenic function. Taken together, these results portray a novel regulatory pathway employing miR-24 and ING5 to fine-tune the balance of breast cancer cells.

Given the involvement of aberrant miRNA expression in the initial and developmental stages of human cancers, therapeutic strategies based on the manipulation of miRNA activity hold great promise to influence cancer behavior. For the anti-tumorigenic miRNAs, induced expression of individual miRNAs, either through transfection or therapeutic delivery methods, has demonstrated the ability to suppress tumor progression without toxicity [27]. Conversely, for cases in which miRNAs are consistently upregulated in cancers, targeting common oncogenic miRNAs through the use of antisense reagents such as antagomirs and locked nucleic acids has proven to be a potential intervention to block cancer-associated miRNAs [28, 29]. In this study, ING5 is downregulated in breast cancer and is negatively regulated by miR-24. Hence, it is quite possible that modulating miR-24 may increase ING5 expression and subsequently activate the anti-tumor effects of ING5. Indeed, restoration of ING5 expression with a miR-24-resistant ING5 overexpression plasmid completely reversed miR-24-induced cellular phenotypes and blocked xenografted tumor growth in vivo. In the future, greater research emphasis is needed to characterize the feasibility of targeting miR-24 in cancer therapy and to develop simplified and cost-effective manipulation methods.

## Conclusions

In conclusion, this study demonstrated that ING5 can suppress the proliferation and invasion of, and stimulate the apoptosis of breast cancer cells. This study also showed for the first time that miR-24 accomplishes its oncogenic effects by negatively regulating ING5 expression in breast cancer. Further research on miR-24 and ING5 may reveal a new avenue for treatment of breast cancer.

## Additional files

Additional file 1: Figure S1. The Spearman’s correlation scatter plot of the fold changes of miR-24 and ING5 protein levels in breast cancer tissue pairs. (TIF 141 kb)

Additional file 2: Figure S2. The effect of miR-24 on the expression of ING5 mRNA and protein after inhibiting transcription with actinomycin D. (A) qRT-PCR analysis of ING5 and c-myc mRNA levels in MCF-7 cells treated with actinomycin D for 8 h. (B-D) qRT-PCR and western blot analysis of ING5 mRNA and protein levels in MCF-7 cells transfected with equal doses of pre-miR-control, pre-miR-24, anti-miR-control or anti-miR-24 followed by treatment with actinomycin D for 8 h. B: qRT-PCR analysis of the ING5 mRNAs levels; C: the representative image of western blot analysis of ING5 protein; D: the quantitative analysis of western blot. **p < 0.01; ***p < 0.001. (TIF 1041 kb)

Additional file 3: Figure S3. Western blot analysis of the expression levels of ING5 protein in MCF-7 cells. (A-B) Western blot analysis of the expression levels of ING5 protein in MCF-7 cells co-transfected with pre-miR-control plus control plasmid, pre-miR-24 plus control plasmid, pre-miR-control plus an ING5-HA overexpressing plasmid, or pre-miR-24 plus an ING5-HA overexpressing plasmid. A: representative image; B: quantitative analysis. UD: undetected; **p < 0.01; ***p < 0.001. (TIF 144 kb)

## Abbreviations

3’-UTRs: 3’-untranslated regions
H&E: Hematoxylin and eosin
ING: Inhibitor of growth
PHD: Plant homeodomain
