# Supplementary figures and images for: ING5 suppresses breast cancer progression and is regulated by miR-24
Source: Mol Cancer. 2017 May 10;16:89. doi: 10.1186/s12943-017-0658-z (PMC5424299; doi:10.1186/s12943-017-0658-z)

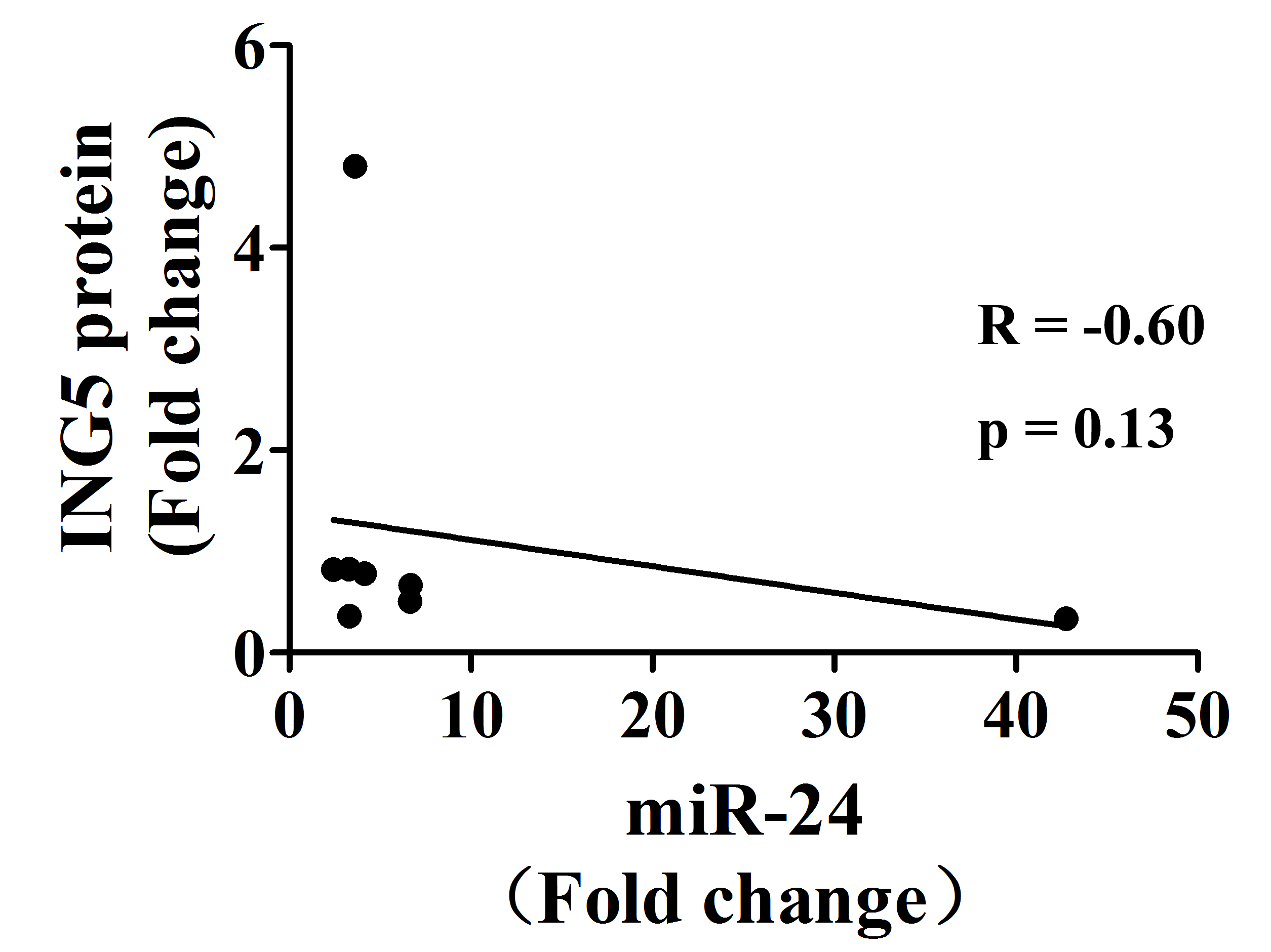

Supplement: Supplementary file 1 — The Spearman’s correlation scatter plot of the fold changes of miR-24 and ING5 protein levels in breast cancer tissue pairs. (TIF 141 kb) [file 12943_2017_658_MOESM1_ESM.tif]

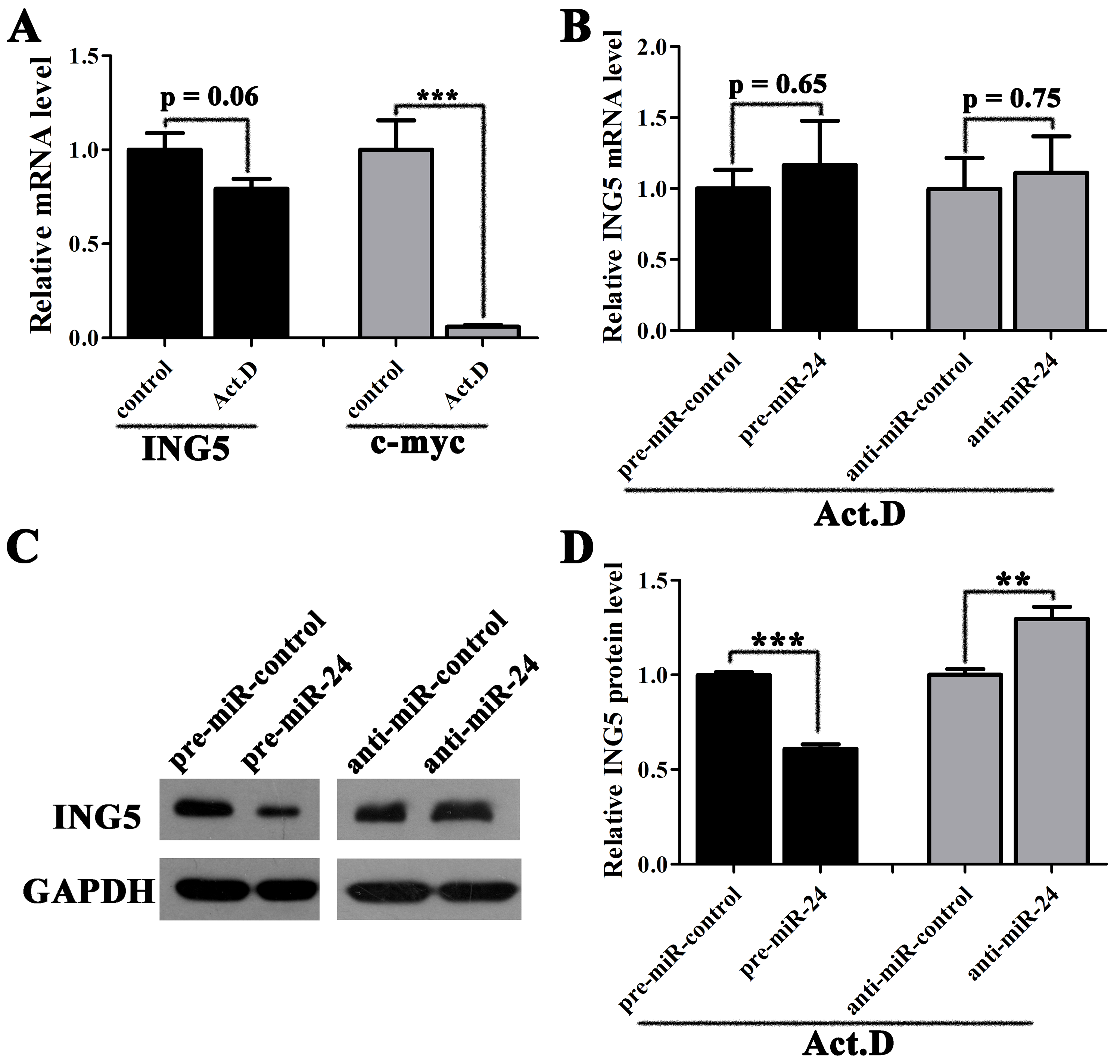

Supplement: Supplementary file 2 — The effect of miR-24 on the expression of ING5 mRNA and protein after inhibiting transcription with actinomycin D. (A) qRT-PCR analysis of ING5 and c-myc mRNA levels in MCF-7 cells treated with actinomycin D for 8 h. (B-D) qRT-PCR and western blot analysis of ING5 mRNA and protein levels in MCF-7 cells transfected with equal doses of pre-miR-control, pre-miR-24, anti-miR-control or anti-miR-24 followed by treatment with actinomycin D for 8 h. B: qRT-PCR analysis of the ING5 mRNAs levels; C: the representative image of western blot analysis of ING5 protein; D: the quantitative analysis of western blot. **p < 0.01; ***p < 0.001. (TIF 1041 kb) [file 12943_2017_658_MOESM2_ESM.tif]

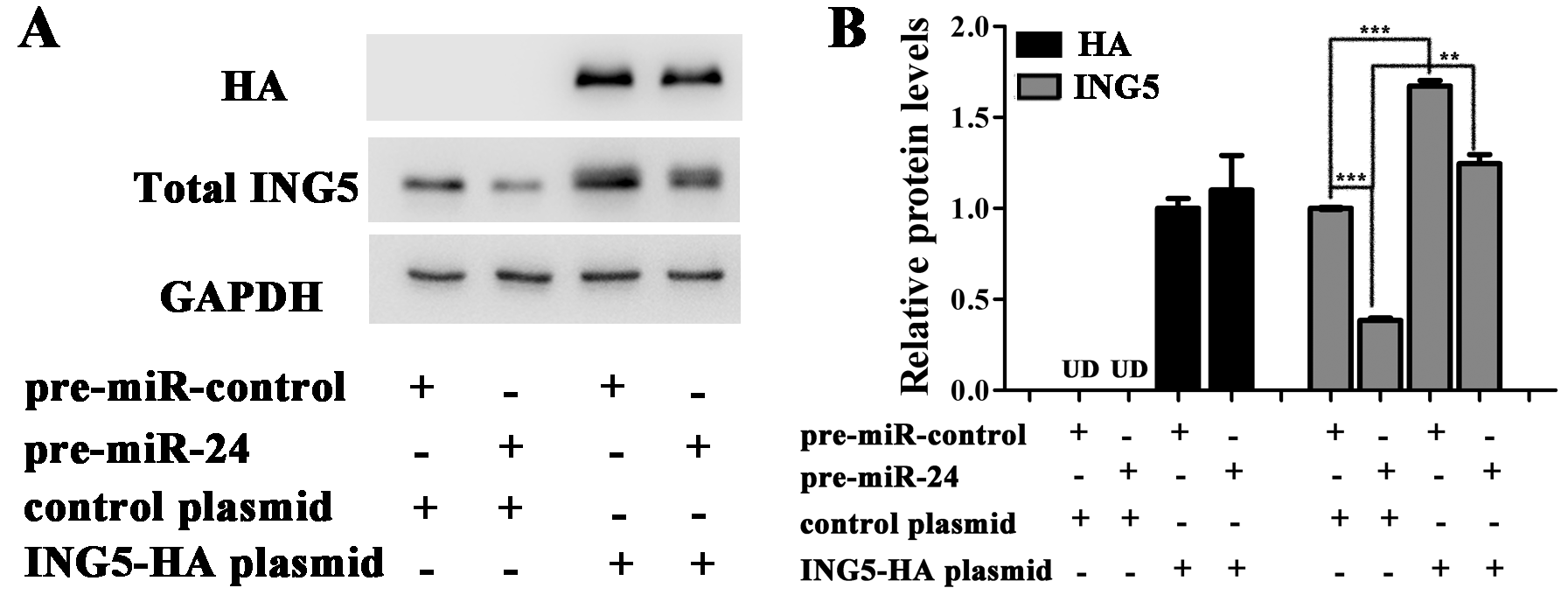

Supplement: Supplementary file 3 — Western blot analysis of the expression levels of ING5 protein in MCF-7 cells. (A-B) Western blot analysis of the expression levels of ING5 protein in MCF-7 cells co-transfected with pre-miR-control plus control plasmid, pre-miR-24 plus control plasmid, pre-miR-control plus an ING5-HA overexpressing plasmid, or pre-miR-24 plus an ING5-HA overexpressing plasmid. A: representative image; B: quantitative analysis. UD: undetected; **p < 0.01; ***p < 0.001. (TIF 144 kb) [file 12943_2017_658_MOESM3_ESM.tif]
